# Dr. Folt’s Case of Audition Restored by the Removal of a Tumor from Hyo-Thyroid Region

**Published:** 1842-12-17

**Authors:** Daniel V. Folts

**Affiliations:** Springville, N. Y.


### MEDICAL EXAMINER.

NEW SERIES.

No. 51.] PHILADELPHIA, DECEMBER 17, 1842. [Vol. I.

### *Audition restored by the Removal of a Tumor from Hyo-Thyroid Region.*

By Daniel V. Folts, M. D., Springville, N. Y.

The patient was a lady in middle life, well marked scrofulous diathesis, and sanguineo-lymphatic temperament. For eight or nine years past, she has suffered more or less inconvenience from a tumour over the larynx, and during the last eighteen months has been deprived, entirely, of the use of the right ear. The history of the case as related by herself is substantially as follows.

In Aug. 1834, she first discovered something over her windpipe, as she has it, about the size of a chestnut. It occasioned but little uneasiness at the time, no pain, and about stationary, as to size, for two or three years. About this time an unnatural beating was discovered in the right ear, and the function of that organ became gradually impaired. The sensation of throbbing was synchronous with the systole of the heart, and by pressure upon the common carotid it would almost entirely cease, and hearing, for the time being, would be much improved. Latterly, however, pressure upon the carotid, has had no other effect than to arrest the throbbing, audition becoming more and more affected—as the tumour increased in size—until for the period above mentioned it has been entirely obliterated. And the fact should not be lost sight of, that during this time the tumour has increased in size more than it had done for the seven preceding years taken together.

At the time I was consulted, the tumour had attained the size of a hen's egg, and in its general outlines not dissimilar to that body, only with a greater difference of longitudinal and transverse diameters. The morbid growth had occasioned a great deal of pain, and for twelve months past had very much impeded deglutition, several efforts being often necessary before the morsel could be swallowed. The details of the operation, contained in my notes of the case, I omit, but that the objects of this paper may be answered, the parts implicated will be hastily enumerated.

The base of the tumour was firmly attached to the os hyoides about the mesial line, and descending obliquely, covered the right fronto-aspect of the thyroid cartilage.

In the dissection, almost the entire basis of the os hyoides was laid bare, and the same may be said of the cutiform cartilage, except its left and inferior margins. Almost the entire mass of the right, together with a small portion of the left hyo-thyroideus muscle was dissected out with the tumour—the muscular fibres being very much degenerated, and nearly lost in the walls of the sac. The oneo-hyoid muscles of both sides were laid bare at their insertions, and a few fibres of the one on the right side were removed with the knife. The ranim vein—in this patient remarkably large—was cut and ligated.

During the dissection on right side of the tumour the patient complained loudly of pain. It was, however, but momentary, and when the operation was completed, she declared she could "hear as well as ever."

In a few days deglutition became easy and natural, the wound healed kindly, and now, several months after the operation, the hearing continues good.

Now that between the growth of the tumour in question, and the gradual loss and final obliteration of hearing in the right ear—the removal of the morbid growth, and the instantaneous recovery of audition, there exists the relation of cause and effect, is a conclusion not easily to be avoided. Well, the facts in the case are stated, the data all given; who will solve, for us, the problem?

